# An Ultra-Precision Smoothing Polishing Model for Optical Surface Fabrication with Morphology Gradient Awareness

**DOI:** 10.3390/mi16070734

**Published:** 2025-06-23

**Authors:** Guohao Liu, Yonghong Deng, Zhibin Li

**Affiliations:** 1 Department of Mechanical and Electrical Engineering, Liaocheng University Dongchang College, Liaocheng 252000, China; liuguohao@lcudcc.edu.cn; 2 Sichuan Provincial Promotion Center of Digital Transformation, Chengdu 611730, China; 3 School of Software Engineering, Chengdu University of Information Technology, Chengdu 610225, China; lizhibin@cuit.edu.cn; 4 Xinjiang Technical Institute of Physics &Chemistry, Chinese Academy of Sciences, Urumqi 830011, China

**Keywords:** ultra-precision optical manufacturing, smoothing polishing, mid-spatial frequency errors, morphology gradient

## Abstract

To improve the surface morphology quality of ultra-precision optical components, particularly in the suppression of mid-spatial frequency (MSF) errors, this paper proposes a morphology gradient-aware spatiotemporal coupled smoothing model based on convolutional material removal. By introducing the Laplacian curvature into the surface evolution framework, a curvature-sensitive “peak-priority” mechanism is established to dynamically guide the local dwell time. A nonlinear spatiotemporal coupling equation is constructed, in which the dwell time is adaptively modulated by surface gradient magnitude, local curvature, and periodic fluctuation terms. The material removal process is modeled as the convolution of a spatially invariant removal function with a locally varying dwell time distribution. Moreover, analytical evolution expressions of PV, RMS, and PSD metrics are derived, enabling a quantitative assessment of smoothing performance. Simulation results and experimental validations demonstrate that the proposed model can significantly improve smoothing performance and enhance MSF error suppression.

## 1. Introduction

During the milling and rough polishing stages of optical component fabrication, MSF errors are often inevitably introduced due to the combined effects of toolontinuous tool paths, non-ideal superposition of material removal functions, and systematic equipment errors. MSF errors lie between low-frequency form errors and high-frequency surface roughness in the spatial frequency domain and have a significant impact on optical imaging performance [1]. In high-performance imaging or laser systems, such errors can lead to small-angle scattering, fringe interference, and other phenomena, ultimately degrading system resolution and contrast. These adverse effects are particularly problematic in several precision-demanding industrial contexts. For instance, in high-energy laser systems, residual MSF errors can induce beam modulation and hot spots, reducing damage thresholds, and impairing energy delivery. In semiconductor lithography optics, mid-frequency ripple structures introduce phase errors that deteriorate pattern fidelity and line-edge precision. Similarly, astronomical telescopes are highly sensitive to MSF-induced wavefront distortion, which compromises imaging contrast and detection capability for faint objects. These examples demonstrate that MSF suppression is not only a theoretical necessity but also a pressing practical requirement across multiple advanced optical engineering domains. Currently, two primary strategies are employed to address MSF errors: suppression and correction. The suppression approach aims to minimize the generation of MSF errors during milling and rough polishing by optimizing process parameters. This includes designing appropriate tool path patterns (e.g., raster, spiral, or pseudo-random paths), optimizing path distribution, and controlling parameters such as pitch and point spacing, thereby avoiding the amplification of specific spatial frequency components. Moreover, maintaining a stable and uniform distribution of the material removal function also helps to reduce process-induced periodic error. The correction approach, on the other hand, targets MSF errors that have already formed. It employs subsequent high-precision processes to selectively remove such errors. Among these, smoothing and polishing a widely adopted and effective methods. By precisely controlling the pressure, trajectory, removal function, and multi-pass processing parameters of flexible polishing tools, this technique suppresses MSF errors and optimizes the spatial frequency distribution of the surface [2]. As a result, the final surface quality is improved to meet the stringent requirements of advanced optical systems.

In recent decades, significant progress has been made in understanding and modeling the smoothing mechanisms in optical surface processing. Early work by Brown and Parks et al. [3] in 1981 introduced a quantitative model for smoothing using flexible abrasive belts supported by elastic substrates. Subsequently, researchers such as Jones et al. [4] proposed simplified linear models to simulate the smoothing process. In 1990, Mehta and Reid et al. [5] developed the classical bridge-type smoothing model grounded in elasticity theory, which later served as a foundation for further refinements. Tuell et al. [6] later enhanced the bridge model by incorporating Fourier decomposition techniques to improve its predictive accuracy. Building upon these earlier contributions, Kim et al. [7] introduced a parametric smoothing model in 2010, extending the bridge-type framework to analyze the smoothing behavior of viscoelastic polishing tools. This model enabled the evaluation of different tool materials and process conditions. In 2013, Shu et al. [8] critically examined Kim’s model, highlighting its assumption of a constant slip factor and pointing out the need to account for the time-dependent nature of the smoothing process. By integrating time-variant characteristics, Shu revealed an exponential decay behavior in surface error evolution, offering deeper insights into the temporal dynamics of smoothing. Further investigations explored the influence of motion strategies and pad designs on smoothing performance. For example, experiments showed that double planetary motion enhances smoothing efficiency by accelerating the decay rate of surface error and reducing the limiting roughness. The introduction of pseudo-random or stochastic tool paths also demonstrated potential in mitigating MSF errors. In parallel, Nie et al. [9] utilized finite element analysis to examine the smoothing of irregular ripple structures, revealing the mechanical response of surfaces to varying tool pressures and geometries. In addition to model development, empirical comparisons have been conducted to assess the effectiveness of different polishing pads. Zhang et al. [10] compared asphalt-based and polyurethane pads under identical operating conditions, finding that the former exhibited superior smoothing capabilities. Kim et al. [11], leveraging their parametric model, concluded that polishing tools made of harder elastic materials tend to produce better smoothing effects. Nie et al. [12] also observed that the design of pad grooves significantly influences material removal characteristics, with radial slotting configurations offering enhanced smoothing outcomes compared to conventional patterns.

However, a critical limitation of the aforementioned models is the lack of consideration for the spatial morphological characteristics of the surface during the smoothing process. Existing approaches typically treat the surface error as a uniform entity and overlook the influence of local gradient variations, curvature distribution, and feature-specific dynamics on material removal behavior. In practical smoothing scenarios, the spatially varying morphology—including peak-valley sharpness and curvature-induced tool-substrate contact differences—plays a crucial role in determining the actual removal efficiency and smoothing outcome. Addressing this limitation requires a model that not only accounts for the temporal evolution of surface error but also integrates spatial morphological feedback into the smoothing strategy.

To this end, this paper proposes a novel Morphology-Gradient-Aware Spatiotemporal Coupled Smoothing Model Based on Convolutional Material Removal. This model incorporates the local gradient magnitude and Laplacian curvature of the surface into the dwell time modulation strategy, establishing a dynamic feedback mechanism that adapts the smoothing intensity based on surface topography. A spatiotemporally coupled control equation is constructed, in which the dwell time is modulated by both spatial and temporal parameters, enabling selective enhancement of removal in regions with sharp morphological features. By integrating the material removal function in a convolutional framework, the model realistically reflects the non-local nature of the smoothing process and allows for high-resolution simulation of surface evolution. The main contributions of this work are summarized as follows:

(a) A novel smoothing framework that integrates surface morphology characteristics—specifically, surface gradient and curvature—into the control of dwell time, enabling topology-aware polishing strategies;
(b) A spatiotemporal nonlinear control model is established, where dwell time modulation is dynamically coupled with surface feature evolution and periodic process perturbations.

The remainder of this paper is structured as follows. Section 2 develops the mathematical foundation and modeling framework. Section 3 presents a detailed simulation analysis. Section 4 conducts the experimental validation. Finally, Section 5 concludes the paper with findings and future directions.

## 2. Theoretical Foundations of Morphology Gradient-Aware Smoothing

### 2.1. Fundamental Principles of Smoothing and Material Removal

**Figure 1:**
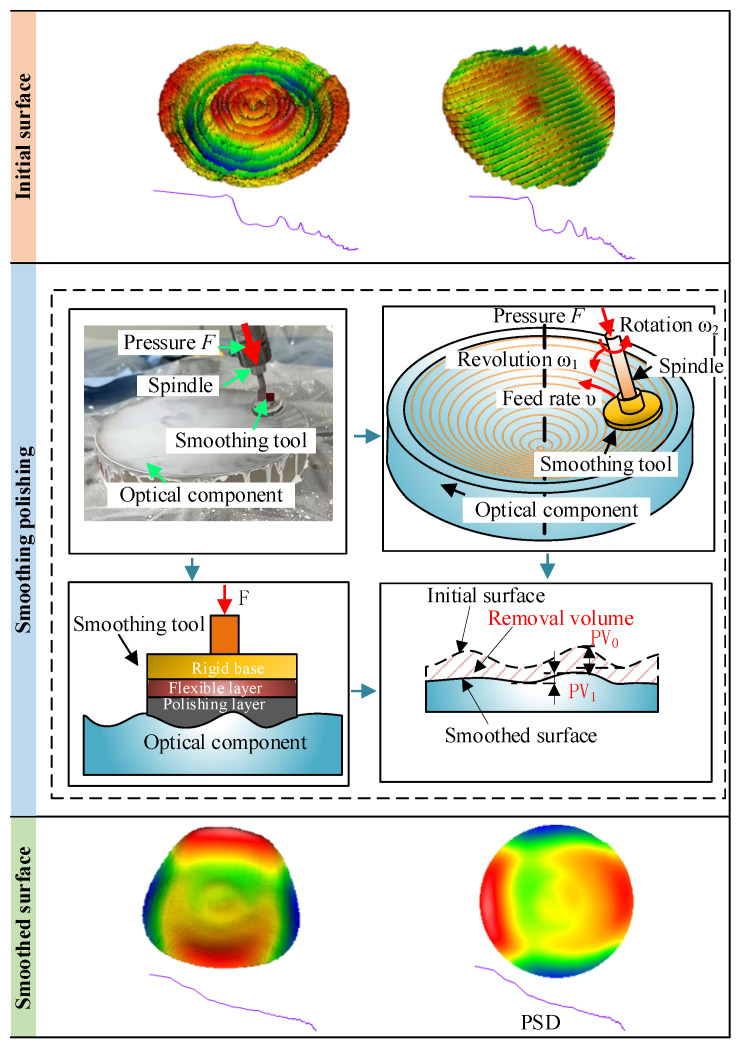
Principle of smoothing polishing. Note that: **Top**: Examples of circular and stripe-like MSF structures on optical surfaces and their corresponding PSD curves. **Middle**: Illustration of the smoothing process using a dual-rotating tool. **Bottom**: Smoothed surfaces.

During optical surface manufacturing, MSF errors are typically introduced by factors such as the superposition of tool paths, discontinuities in machining path planning, and system-related equipment errors [13]. Figure 1 schematically illustrates the basic principle of smoothing-based correction for MSF errors.

This error can manifest in various forms, including periodic undulations with certain regularity, such as rings or stripes, as well as random undulations without clear periodicity. Figure 1 presents examples of MSF errors with circular and striped features. However, in practical engineering applications, the morphology of MSF errors can be far more complex and diverse, encompassing various periodic and non-periodic tool path traces. A widely adopted strategy for correcting MSF errors involves optimizing the grinding tool design and process parameters, followed by implementing a smoothing polishing process. Typically, a dual-rotating polishing tool combining rotational and orbital motions is employed to achieve uniform smoothing across the optical surface. The effectiveness of this process relies on two key conditions: first, the polishing tool must possess sufficient rigidity to ensure stable transmission of polishing pressure; second, the tool diameter must be large enough to effectively cover the spatial wavelength range of the target error. During smoothing, the surface high points come into preferential contact with the polishing tool and bear higher localized pressure, leading to a higher material removal rate compared to the low points. This creates a dynamic mechanism favoring the removal of high points. Overall, the smoothing process acts as a low-pass filter on the optical surface morphology, effectively attenuating the error amplitudes within the MSF band. Following smoothing treatment, the optical surface exhibits a more uniform and smooth morphology, with a significant reduction in the amplitude of mid-frequency peaks in the power spectral density (PSD) curve. The general procedure for full-aperture uniform polishing correction consists of the following steps: first, detecting the initial surface error through high-precision shape measurement; second, performing PSD analysis to identify MSF errors exceeding acceptable thresholds; third, selecting appropriate grinding tool parameters and process conditions based on the analysis; and finally, optimizing the smoothing process to progressively reduce MSF errors to meet the specified design requirements.

**Figure 2:**
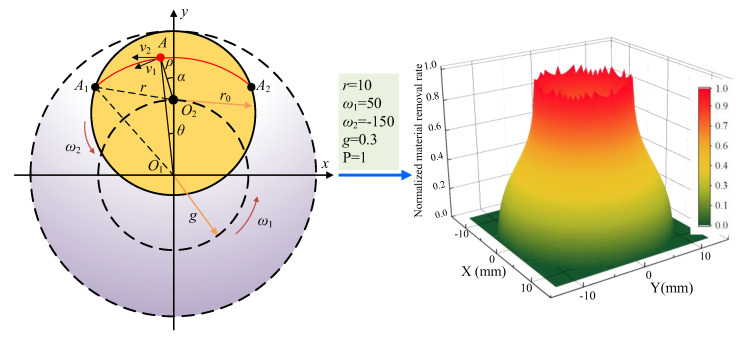
Motion analysis of the smoothing tool and its material removal function.

The kinematic relationship of the dual-rotating smoothing tool is illustrated in Figure 2. Here, *O*_1_ denotes the center of revolution, and *O*_2_ represents the center of rotation, which is also the center of the smoothing tool.

Based on Preston’s theory, the distribution of material removal within the processed region of the optical component can be characterized by a material removal function [14]:

(1) $$\begin{array}{l} R(r)=\frac{Kp\omega_{1}}{2\pi }{\int_{-\theta_{0}}^{\theta_{0}}\left[r^{2}{\left(1+f\right)}^{2}+r_{0}^{2}f^{2}e^{2}-2rr_{0}fe(1+f)\cos\theta \right]}^{1/2}d\theta ,\\ \theta_{0}=\arccos(\frac{r^{2}+(e^{2}-1)r_{0}^{2}}{2rer_{0}}),f=\omega_{2}/\omega_{1},\\ e=g/r_{0},r\in [0,(1+e)r_{0}] \end{array}$$

where *g* denotes the eccentricity, representing the revolution radius; *r*_0_ is the radius of the smoothing tool; *ω*_1_ and *ω*_2_ are the angular velocities of the revolution and rotation motions, respectively. The total effective coverage radius of the tool’s removal function is therefore *g* + *r*_0_.

Figure 2 presents a practical example of a removal function and lists the corresponding parameters in detail.

### 2.2. Morphology Gradient Aware Spatiotemporal Coupled Model

#### 2.2.1. Instantaneous Material Removal Modeling Driven by Surface Topography

The surface error of the optical component is modeled using a superposition of sinusoidal and cosinusoidal functions, expressed as:

(2) $$z(x,y)=\sum_{i=1}^{N}A_{i}\sin(\frac{2\pi x}{\alpha_{i}})+\sum_{j=1}^{M}B_{i}\cos(\frac{2\pi x}{\beta_{i}})+\sum_{k=1}^{Q}C_{i}\cos(\frac{2\pi x}{\lambda_{i}}),$$

where *z*(*x*, *y*) denotes the surface error at location (*x*,*y*), *A_i_*, *B_i_* and *C_i_* are the amplitude coefficients, respectively. *α_i_*, *β_i_*, and *λ_i_* are the spatial wavelengths corresponding to each directional component, respectively.

It is assumed that the smoothing tool is uniformly pressed downward by a fixed displacement *z*_0_. Given the relatively small thickness of the flexible layer, it can be modeled as a Winkler foundation (i.e., an elastic substrate). Under this assumption, the resulting pressure distribution on the optical surface can be expressed as follows:

(3) $$p(x,y)=\left\{\begin{array}{c} k\cdot \delta (x,y),\delta (x,y)>0\\ \;\;\;0,\;\;\;\delta (x,y)\leq 0 \end{array}\right.,$$

where *δ*(*x*, *y*) represents the compression of the smooth tool, and *k* is the stiffness.

The local compressive deformation *δ*(*x*, *y*) between the tool and the optical surface is:

(4) $$\delta (x,y)=\max\left\{0,z_{0}-z(x,y)\right\}.$$

The maximum function ensures that only contact points contribute to pressure; non-contact areas yield zero pressure.

In smoothing polishing modeling, the material removal function *R*(*x*, *y*) is defined as the spatial distribution of material removed by the tool per unit time. In conventional models, *R*(*x*, *y*) is often assumed to be spatially invariant, representing the tool’s removal capability on an ideally flat surface. However, in real-world scenarios, the actual contact between the tool and the workpiece surface is affected by local topography, resulting in spatially varying compression *δ*(*x*, *y*). According to the contact mechanics model described in Equation (3), the local pressure *p*(*x*, *y*) is linearly proportional to the compression *δ*(*x*, *y*). Following the classical Preston equation, the local material removal rate is proportional to the product of pressure and relative velocity. As the relative velocity between the tool and the surface is treated as a constant parameter in this study, the removal rate becomes directly proportional to the local pressure. Consequently, the actual material removal function should be modulated by the compression field. Thus, the originally assumed uniform removal function *R*(*x*, *y*) fails to account for this compression-induced variation, which may lead to prediction errors. To address this, a compression-modulated mechanism is introduced, in which the true removal function *R*′(*x*, *y*) is expressed as:

(5) $$R\prime (x,y)=C\cdot R(x,y)\cdot \max(0,k(z_{0}-z(x,y))),$$

where *C* is a normalization constant to ensure the total material removal value *MR_total_*. *R*(*x*, *y*) denotes the intrinsic material removal function.

(6) $$MR_{total}=\iint_{\Omega }R\prime (x,y)dxdy=\iint_{\Omega }C\cdot R(x,y)\cdot \max(0,k(z_{0}-z(x,y)))dxdy$$

The numerical calculation of Equation (6) is as follows:

(7) $$MR_{total}=\sum_{i=1}^{N}\sum_{j=1}^{M}R\prime (x_{i},y_{i})\cdot \Delta x\cdot \Delta y.$$

By applying Equation (7), the pressure distribution over the surface topography can be accurately obtained. This pressure field reflects the localized contact stress induced by the smoothing tool under displacement-controlled loading. Based on the computed pressure distribution, the instantaneous material removal rate at any point on the optical surface can be further determined, enabling the estimation of the spatial distribution of material removal within a given region. This provides a quantitative foundation for predicting smoothing effectiveness and guiding dwell time optimization.

**Figure 3:**
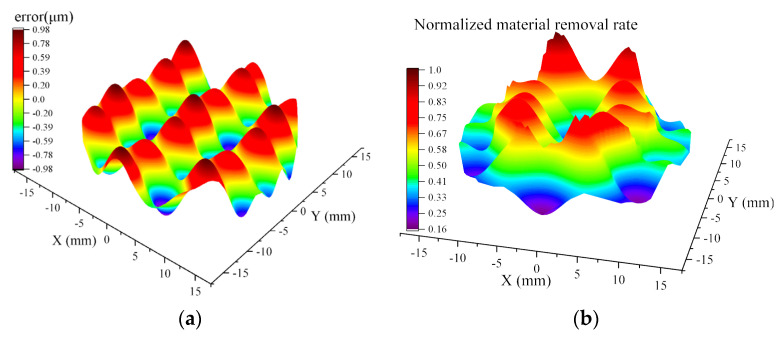
Simulation of material removal over a surface with MSF errors. (**a**) Synthetic surface error profile; (**b**) Instantaneous material removal distribution.

Figure 3 illustrates a representative simulation example. As shown in Figure 3a, a synthetic surface error map is defined using a superposition of trigonometric functions to emulate MSF errors. Under a given external load *F*, the corresponding pressure distribution is computed based on the topographical features and the tool influence function (TIF) as described previously. Using this pressure field, the instantaneous material removal distribution over the smoothing region is obtained, as depicted in Figure 3b. This result demonstrates the coupled influence of surface error morphology and the pressure attenuation characteristics of the tool and serves as a basis for evaluating the localized smoothing response.

Although the current model employs a linear pressure–deformation relationship inspired by the Winkler elastic foundation theory, which assumes independent vertical displacement at each surface point, it is acknowledged that real-world polishing processes may involve more complex physical interactions. These include viscoelastic effects of the pitch pad, thermal-induced softening, and fluid-mediated pressure redistribution due to slurry flow. Nonetheless, under the controlled conditions typical of ultra-precision smoothing—characterized by low material removal rates and small deformation—the linear assumption remains a valid and widely accepted approximation. Moreover, by integrating the Winkler model with surface morphological descriptors—specifically, local gradient and curvature—the removal function effectively captures the influence of surface topography on compression behavior. This approach enhances the physical realism of the model while maintaining computational simplicity. For broader applicability, future research could incorporate more advanced foundation models, such as Pasternak-type or viscoelastic systems, and consider fluid–structure interaction under high-load or high-speed polishing scenarios.

#### 2.2.2. Modeling Framework Based on Surface Morphology Gradient

To enable more efficient and adaptive smoothing control of optical surfaces, a Morphology Gradient Aware Spatiotemporal Coupled Smoothing Model is proposed. This model integrates surface residual gradient features, curvature factors, a periodic perturbation modulation mechanism, and the convolution principle of the removal function. It is capable of dynamically adjusting dwell time and enabling region-specific material removal control.

Let *M*(*x*, *y*) denote the actual material removal at each point on the surface, and *Z*′(*x*,*y*) represent the post-processed surface profile. The goal of the proposed model is to achieve intelligent regulation of the dwell time distribution *T*(*x*, *y*), ensuring that the final surface *Z*′(*x*,*y*) satisfies the target form accuracy specifications. In optical surface processing, the amount of material removed is conventionally described by a convolution model that relates dwell time and the removal function:

(8) M(x,y)=∬R(x−ξ,y−η)T(ξ,η)dξdη

where *M*(*x*, *y*) denotes the actual material removal, *R*(*x*, *y*) represents the removal function. *T*(*ξ*, *η*) is dwell time distribution.

To achieve precise perception and control of the evolution of surface morphology during the smoothing process, it is essential to introduce gradient and curvature-based descriptors that characterize the local topography. These descriptors serve as sensitivity indicators to capture the dynamic changes in slope and curvature across the surface, thereby enabling adaptive modulation of material removal strength based on regional surface features. Specifically, the surface gradient reflects the rate of local elevation change, while the Laplacian operator characterizes the degree of concavity or convexity. Incorporating these geometric features allows for spatially differentiated control during smoothing, enhancing the model’s ability to suppress MSF errors and improve surface uniformity.

(9) $$\left|\nabla z(x,y)\right|=\sqrt{{\left(\frac{\partial z(x,y)}{\partial x}\right)}^{2}+{\left(\frac{\partial z(x,y)}{\partial y}\right)}^{2}},$$

where |∇Z| represents the local surface slope magnitude, which quantifies the rate of elevation change across the surface at each point. It serves as a sensitive indicator of local error intensity, allowing for the identification of steep regions where material removal should be prioritized. The higher the gradient, the sharper the feature, which implies a greater contribution to MSF errors. Therefore, incorporating |∇Z| into the smoothing strategy enables morphology-aware, high-priority correction of steep surface anomalies.

The second-order Laplacian operator is calculated as follows:

(10) $$\nabla^{2}z(x,y)=\frac{\partial^{2}z(x,y)}{\partial x}+\frac{\partial^{2}z(x,y)}{\partial y}.$$

The second-order Laplacian operator is used to characterize the local curvature profile of the surface. Specifically, it quantifies the degree of convexity or concavity by summing the second partial derivatives of the surface height in the x and y directions. A positive Laplacian value typically corresponds to a concave region, while a negative value indicates a convex peak. This geometric descriptor plays a critical role in identifying localized surface features such as ridges or depressions that require targeted smoothing.

At each toolpath point (*x*_*k*_, *y*_*k*_), a local neighborhood region *D*_*k*_ is constructed to calculate the average values of gradient magnitude and Laplacian curvature. These metrics, denoted as *G*_*k*_ and *L*_*k*_ respectively, reflect the local surface slope variation and curvature intensity. They serve as key indicators for morphology-aware dwell time modulation. High-gradient or high-curvature regions typically indicate localized defects or abrupt shape transitions, thus requiring greater smoothing emphasis during the process.

(11) $$\left\{\begin{array}{l} G_{k}=\frac{1}{N_{k}}\underset{(x,y)\in D_{k}}{\sum \left|\nabla z(x,y)\right|}\\ L_{k}=\frac{1}{N_{k}}\underset{(x,y)\in D_{k}}{\sum \left|\nabla^{2}z(x,y)\right|} \end{array}\right.,$$

where *G_k_* represents the degree of undulation of the topography around the point, and *L_k_* reflects the intensity of its local curvature.

To modulate the dwell time adaptively based on surface features, a modulation factor *γ*_*k*_ is introduced at each toolpath point:

(12) $$\gamma_{k}=clip(a_{0}+a_{1}G_{k}+a_{2}L_{k},\gamma_{\min},\gamma_{\max}),$$

where *γ*_*k*_ is a modulation factor, *a*_0_, *a*_1_, and *a*_2_ are empirical coefficients corresponding to the baseline, surface gradient, and curvature intensity, respectively. The function *clip*(·) constrains the modulation result within a bounded interval defined by *γ*_min_ and *γ*_max_, thereby avoiding extreme values and enhancing the robustness of the model under varying morphological conditions. Formally, the operator is defined as:

(13) $$clip(x,\gamma_{\min},\gamma_{\max})=\min(\max(x,\gamma_{\min}),\gamma_{\max}),$$

where *x* is the computed modulation value prior to bounding. This ensures that the dwell time scaling factor remains within a physically and numerically stable range.

The selection of empirical coefficients in Equation (12) is based on both practical polishing considerations and component-specific simulation analysis. The parameter ranges are defined to ensure stability and adaptive responsiveness during the smoothing process. Specifically, *a*_0_ provides a baseline dwell time and is chosen from [0.5, 1.5] to ensure minimal removal in flat regions. Coefficients *a*_1_ and *a*_2_, ranging from [0.5, 1.2] and [0.2, 0.8], respectively, control the sensitivity to local surface gradient (*G_k_*) and curvature variation (*L_k_*). To prevent excessive or insufficient material removal, the modulation factor *γ_k_* is constrained within [0.5, 2.0] using clipping bounds γ_min_ and *γ*_max_. To determine the optimal coefficient values for a specific component, simulation experiments are conducted to evaluate the convergence performance under various parameter combinations. For Component 1, the coefficients were optimized using the particle swarm optimization (PSO) algorithm, aiming to minimize the residual RMS and PV value while maintaining surface uniformity. The resulting optimal set of parameters was:

*a*_0_ = 1.0, *a*_1_ = 0.9, *a*_2_ = 0.6, γ_min_ = 0.5, γ_max_ = 2.0. This coefficient set was then used in the morphology-aware dwell time modulation strategy presented in Section 3 to achieve high-precision smoothing performance.

To simulate the smoothing system disturbance, a disturbance term is added:

(14) $$T_{k}=\gamma_{k}T_{0}+A(\frac{2\pi k}{T_{period}})\cdot (2\cdot rand-1),$$

where *T*_0_ denotes the baseline dwell time, which represents the uniform smoothing intensity in the absence of morphological modulation. *A* defines the amplitude of the periodic disturbance introduced to mimic tool dynamics or federate fluctuations. *T_period_* controls the frequency of this sinusoidal perturbation, allowing the simulation of cyclic processing effects that may arise from spindle rotation, tool path periodicity, or environmental oscillations during the smoothing process. *rand* represents a uniformly distributed random scalar in the range [0, 1].

To enhance the realism of the modulation strategy, a periodic disturbance term is introduced in Equation (14). This term is intended to simulate the small-scale, quasi-periodic fluctuations that commonly occur during actual polishing operations. Such fluctuations may stem from path overlap patterns, rotational motions of the tool, or mechanical vibrations in the system. Previous studies have highlighted that these disturbances often influence the distribution of residual MSF errors and may even lead to periodic artifacts if not properly mitigated. The sinusoidal modulation serves to mimic this behavior in a controlled manner, thereby improving the frequency-domain smoothness of the final surface. Similar modeling approaches have been employed in prior research to characterize and suppress MSF formation during sub-aperture polishing processes [15,16].

Since the instantaneous removal function *R*’(*x*, *y*) varies spatially due to pressure field modulation and morphological response, it no longer satisfies the space invariance assumption required by the classical convolution model. Therefore, the overall material removal must be expressed as a discrete summation over all toolpath points, where the local dwell time and position-dependent removal kernel are jointly considered. The revised model is given by:

(15) $$M\prime (x,y)=\sum_{k=1}^{N}R_{k}\prime (x-x_{k},y-y_{k})\cdot T_{k}$$

This formulation captures the localized, nonuniform interaction between the tool and the surface, and provides a more physically realistic framework for surface evolution under morphology aware smoothing control. It is important to note that Equation (8) assumes a spatially invariant removal function, where the same tool influence function is convolved with the dwell time distribution across the surface. By contrast, Equation (15) performs a spatially varying discrete convolution, where the removal function *R*_*k*_′ at each toolpath point is adapted according to local surface morphology and pressure modulation, enabling a more realistic modeling of nonuniform tool–surface interactions.

## 3. Simulation for Model Validation

**Figure 4:**
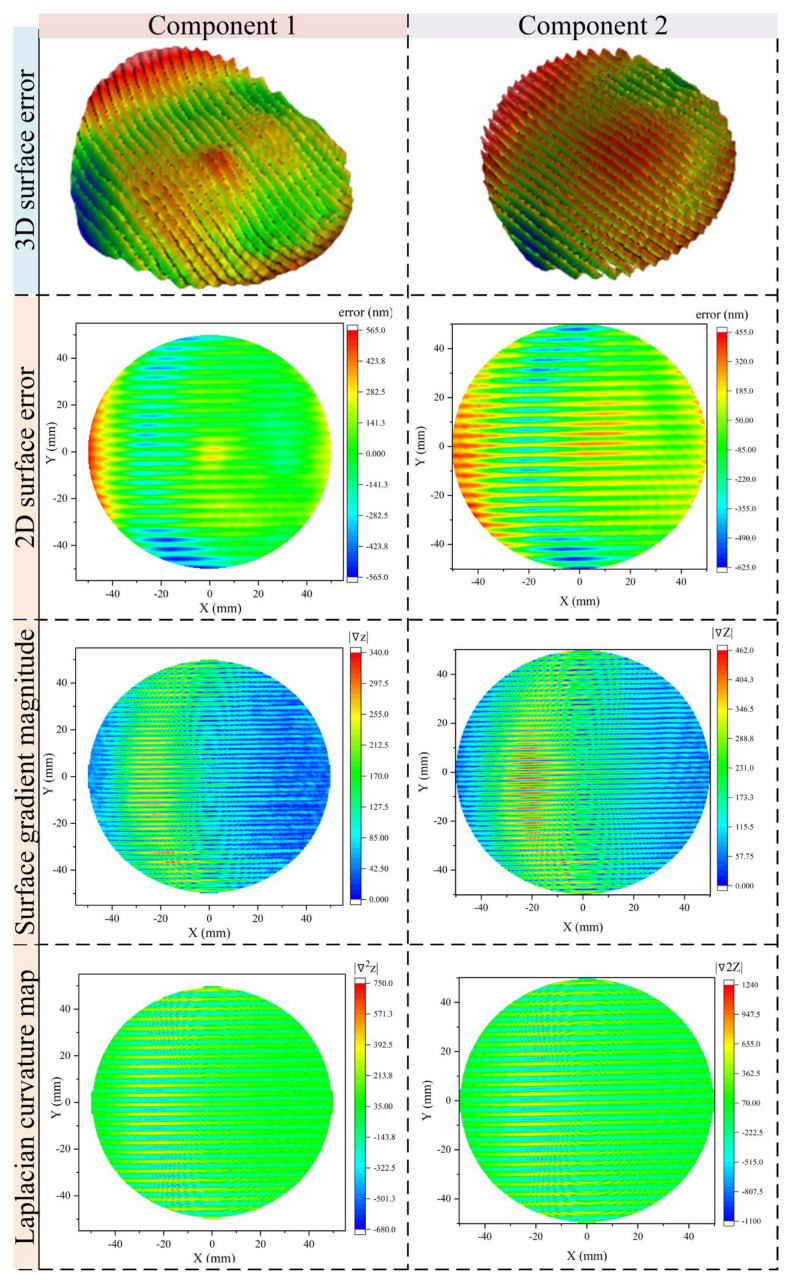
Characterization of optical surface error morphology.

To evaluate the effectiveness of the proposed morphology gradient aware spatiotemporal coupled smoothing model, simulations were performed using real surface measurement data obtained from two optical components. These surfaces exhibit typical MSF errors patterns commonly encountered after deterministic polishing or sub-aperture finishing. By applying the proposed model to actual measured morphology, the simulation aims to validate the model’s ability to guide morphology-aware dwell time control and accurately simulate material removal behavior. Figure 4 illustrates the detailed surface morphology characterization for the two components. For each sample, four representative maps are presented: a three-dimensional rendering of the surface error distribution, a two-dimensional top view of the surface profile, the spatial distribution of surface gradient magnitude, and the Laplacian curvature map of the error surface. These maps provide both geometric and differential perspectives of the measured surface, which are critical for assessing the sensitivity of the model to local topographic variations and validating the role of gradient and curvature in the smoothing response.

In the subsequent simulation, Component 1 is selected to validate the proposed morphology gradient aware spatiotemporal coupled smoothing strategy. The simulation compares two approaches: (1) the proposed method, which adaptively modulates the dwell time at each toolpath point based on surface gradient and curvature information, and (2) a conventional smoothing strategy with constant dwell time applied uniformly along the path, serving as the baseline. Both simulations are conducted using the same tool geometry, path trajectory, and pressure settings. The only distinction lies in the dwell time distribution strategy. In the proposed method, the dwell time is locally adjusted according to the surface morphology, aiming to enhance removal in high-slope or high-curvature regions while conserving time in flatter zones.

**Figure 5:**
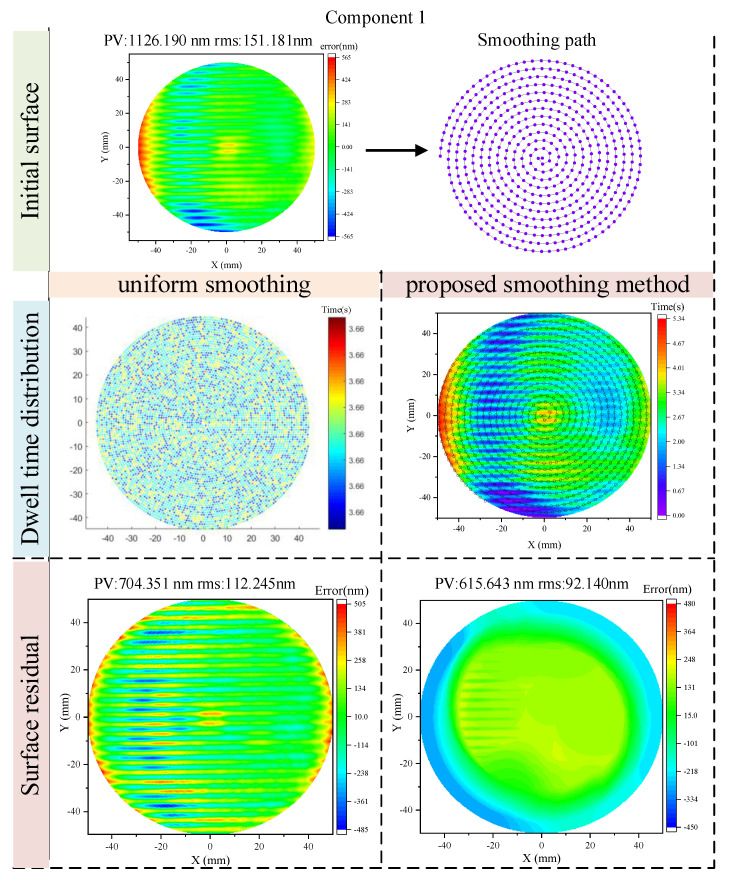
Comparison of smoothing results for component 1 under two strategies.

Figure 5 presents a comparative simulation of the smoothing results for Component 1 using two different strategies: the baseline uniform smoothing method and the proposed morphology-aware smoothing approach. The top row shows the initial surface morphology and the spiral toolpath used in both cases. The middle row illustrates the resulting dwell time distributions for each strategy. In the uniform case, dwell time is applied evenly across all points, leading to a spatially random distribution. In contrast, the proposed method adaptively allocates dwell time according to the local surface slope and curvature, resulting in a highly structured and targeted distribution. The bottom row displays the resulting surface residuals after smoothing. The uniform strategy yields a residual surface with noticeable MSF patterns, achieving a PV of 704.351 nm and an RMS of 122.245 nm. In comparison, the proposed method significantly improves smoothing effectiveness, with the residual PV reduced to 615.643 nm and RMS to 92.14 nm. The smoother spatial distribution and lower residual values confirm the advantage of morphology-driven dwell time modulation in suppressing localized error regions and improving overall surface quality.

## 4. Experimental Validation

### 4.1. Experimental Setup

To further verify the practical applicability of the proposed morphology gradient-aware spatiotemporal coupled smoothing model, an experimental validation was conducted on a precision optical component. While simulation results demonstrate the model’s capability to suppress MSF errors under idealized conditions, experimental testing is essential to evaluate its performance in real-world scenarios involving system uncertainties, material heterogeneity, and tool-surface interactions. The experiment was designed to replicate the smoothing conditions assumed in the model, including tool geometry, path trajectory, and pressure settings. Two smoothing strategies were compared: the conventional uniform dwell-time polishing and the proposed morphology-aware dwell time modulation method.

**Figure 6:**
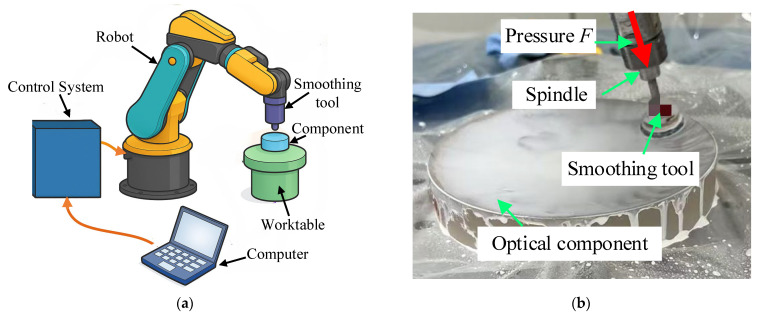
Experimental setup of the smoothing polishing process. (**a**) schematic diagram of the robotic smoothing system; (**b**) actual experimental scene.

Figure 6 illustrates the experimental setup for the smoothing polishing process. Figure 6a presents a schematic diagram of the robotic polishing system, which consists of a six-axis robotic arm, a polishing head with a pitch-based tool, a worktable for fixing optical components, a computer for path planning and data processing, and a control system for synchronized motion and force regulation. Figure 6b shows an actual experimental scene, where a polishing spindle applies controlled pressure onto the optical component surface through the flexible smoothing tool. The polishing motion follows a spiral path to achieve full-area coverage across the workpiece. A CeO_2_-based slurry is used to facilitate material removal. As detailed in Table 1, the experimental setup was built upon a six-axis industrial robot systemintegrated with a custom-designed polishing end-effector. The polishing head employs a pitch pad structure mounted on a rigid aluminum backing. To ensure compliance with the surface, a sponge buffer layer is adhered to the rigid base, followed by a 2–3 mm thick pitch layer as the working surface. This layered configuration enhances contact conformity while preserving local stiffness during smoothing operations. The polishing slurry is prepared using cerium oxide (CeO_2_) powder dispersed in deionized water. No strict mass ratio is enforced; the concentration is adjusted to ensure stable flow and uniform suspension during the process. All experiments are conducted in a clean, temperature-stable environment (25 °C). all processing parameters—including motion strategy, environmental conditions, and tool specifications—are systematically regulated to ensure process repeatability.

### 4.2. Results Analysis

To evaluate the smoothing performance of the proposed morphology gradient aware spatiotemporal coupled smoothing model, surface profiles of the optical components were acquired both before and after the smoothing process using a high-resolution non-contact 3D optical profilometer. This measurement technique ensures high fidelity in capturing the fine-scale topographical features of the optical surface without introducing additional mechanical interference. The acquired surface data were then subjected to comprehensive quantitative analysis by extracting key surface quality indicators, including PV, RMS roughness, and PSD. These metrics represent, respectively, the maximum height variation across the surface, the overall energy of the surface residual error, and the frequency-dependent distribution of surface errors. Together, they provide a multi-dimensional evaluation framework to assess the effectiveness of surface error suppression, particularly in the MSF range, which is critical for the optical performance of high-precision components. This analytical approach allows a clear comparison between the conventional uniform smoothing method and the proposed morphology-driven adaptive smoothing strategy.

(1) Surface Error Evolution Before and After Smoothing.

**Figure 7:**
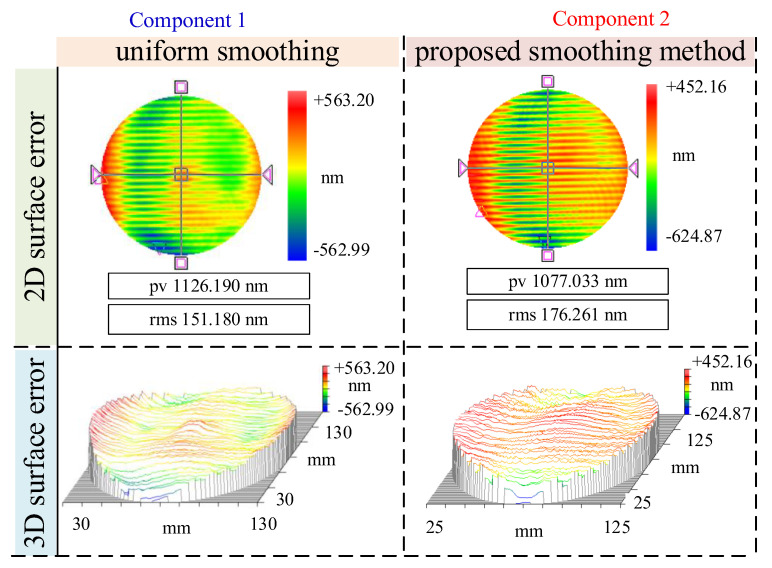
Comparison of initial surface error.

**Figure 8:**
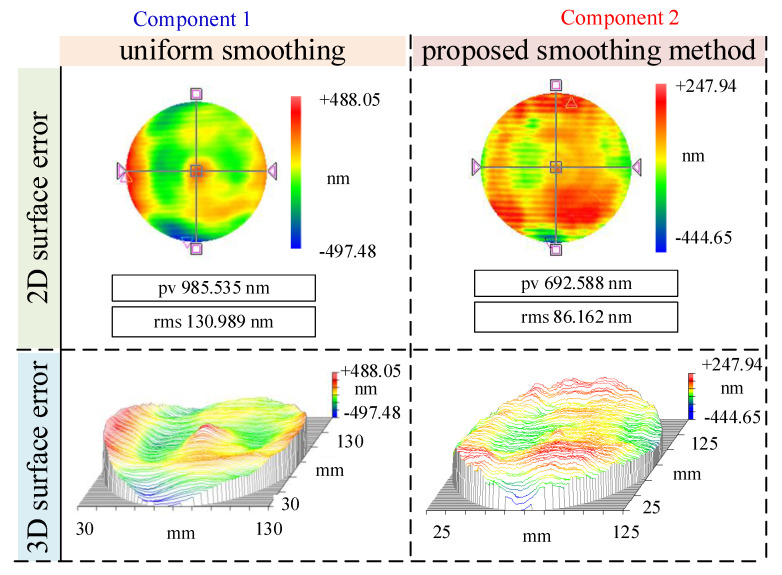
Comparison of after smoothing surface error.

As shown in Figure 7 and Figure 8, both Component 1 and Component 2 initially exhibited prominent surface irregularities, primarily characterized by periodic ripples and band-like structures—typical signatures of MSF errors introduced during prior milling or pre-polishing operations. These errors were particularly evident in the 2D surface maps and further emphasized in the 3D topographies, which revealed ridge-valley alternations aligned along specific directions. Such features can significantly deteriorate optical performance by inducing stray light and scattering effects. After applying the conventional uniform smoothing method, some degree of surface improvement was achieved, as evidenced by the slight reduction in PV and RMS values. However, residual errors remained spatially nonuniform, and the characteristic ripple patterns persisted to a considerable extent. This highlights the limited capability of uniform smoothing in addressing structured or directionally biased error distributions, as it lacks the spatial adaptiveness required to selectively suppress high-gradient or high-curvature regions. In contrast, the proposed morphology gradient-aware spatiotemporal coupled smoothing method resulted in a marked transformation of the surface morphology. The periodic ripples were significantly attenuated, and the remaining residuals appeared more randomly distributed and isotropic. The 3D plots illustrate smoother, more uniform surfaces with diminished slope transitions and a clear suppression of directional error trends. This demonstrates the proposed model’s effectiveness in identifying and prioritizing regions requiring greater smoothing emphasis, particularly in areas with pronounced gradient or curvature features.

(2) Quantitative Evaluation of PV and RMS Indicators

**Figure 9:**
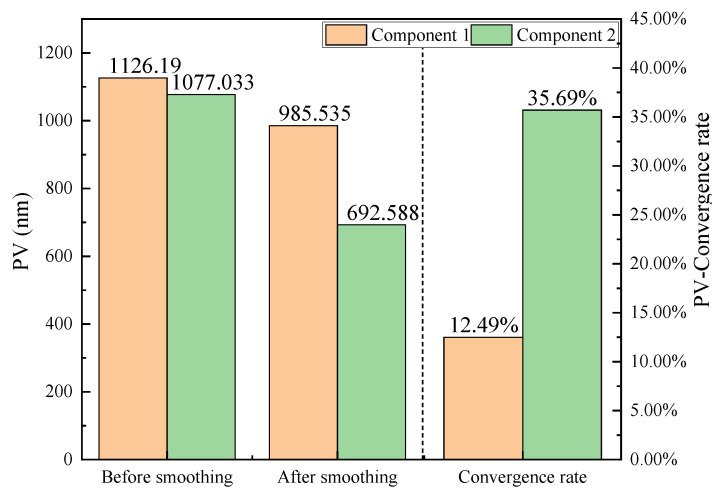
PV evolution comparison.

**Figure 10:**
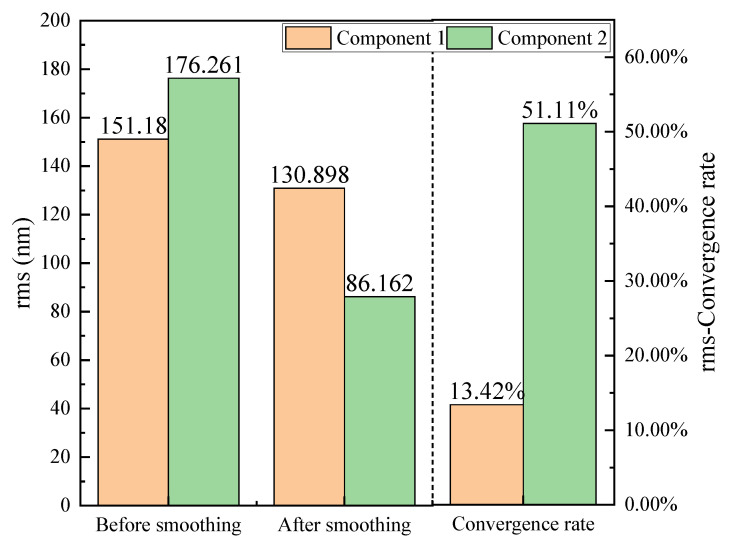
rms evolution comparison.

To directly compare the baseline method and the proposed strategy. Figure 9 and Figure 10 present a comparative analysis of the PV and RMS metrics before and after smoothing, offering a quantitative assessment of the error suppression capabilities of the two approaches. For Component 1, which underwent traditional uniform smoothing, the PV value showed a modest reduction from 1126.19 nm to 985.54 nm, corresponding to a convergence rate of 12.49%. Similarly, the RMS value decreased by only 13.42%. In stark contrast, Component 2—processed using the proposed method—demonstrated significantly greater improvements. The PV dropped from 1077.03 nm to 692.59 nm, representing a convergence rate of 35.69%, while the RMS decreased sharply from 176.26 nm to 86.16 nm, yielding a convergence rate of 51.11%. These metrics indicate a much higher material removal selectivity and precision when the dwell time is adaptively modulated based on surface gradient and curvature descriptors. This validates the superiority of the proposed method over the baseline in terms of convergence performance for both PV and RMS error metrics.

The improved convergence rates observed in Component 2 highlight not only the effectiveness of the proposed method in reducing overall error amplitudes but also its enhanced convergence rate in spatial error correction. By targeting regions with steeper slopes and higher local curvature, the model dynamically adjusts smoothing emphasis, thereby accelerating convergence toward a more uniform surface. Moreover, this adaptability underscores the robustness of the proposed model in responding to diverse surface features and improving deterministic control over the smoothing process.

(3) Frequency Domain Analysis via PSD Curves

**Figure 11:**
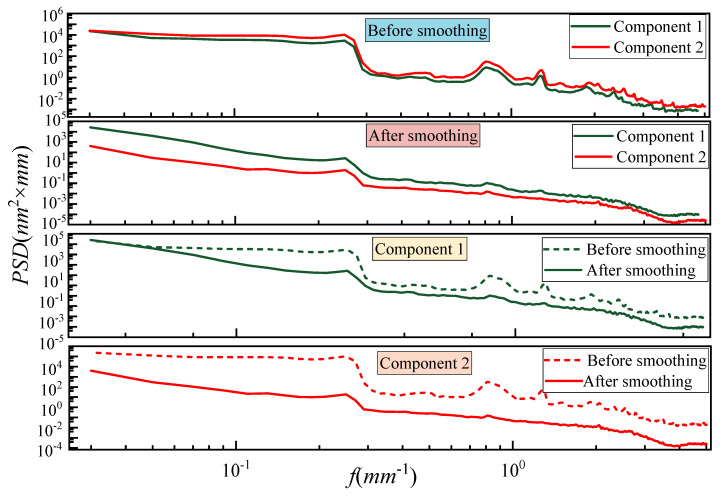
Comparison of PSD curve evolution before and after smoothing.

Figure 11 illustrates the evolution of PSD curves for Components 1 and 2 before and after smoothing, providing an in-depth evaluation of frequency-domain surface quality. Prior to smoothing, both components exhibited elevated PSD amplitudes across the entire spatial frequency range, especially within the MSF band (~0.1–1.0 mm^−1^). After processing, a notable divergence in smoothing performance is observed between the two methods. The uniformly smoothed Component 1 shows only a modest reduction in PSD amplitude, with residual mid-frequency structures still prominent, indicating insufficient suppression of periodic surface errors. This reflects the inability of uniform dwell strategies to accommodate spatially varying surface features, resulting in limited correction of localized error peaks. In contrast, the proposed strategy applied to Component 2 demonstrates a substantial reduction in PSD magnitude over a wide frequency spectrum. Most prominently, the PSD amplitude within the MSF range (0.1–1.0 mm^−1^) drops by nearly an order of magnitude, revealing the model’s superior capacity to modulate material removal in a frequency-selective manner. Additionally, the post-smoothing PSD curves for Component 2 exhibit a more gradual and uniform decay, indicative of enhanced surface isotropy and reduced spatial correlation in residual error features. This suggests that the proposed method not only suppresses dominant frequency peaks but also improves the overall frequency balance of the surface texture. Collectively, the PSD analysis confirms that the proposed model achieves more effective mid-frequency error correction and delivers superior surface uniformity in both spatial and spectral domains.

The experimental results confirm the effectiveness of the proposed morphology gradient-aware spatiotemporal coupled smoothing model in suppressing MSF surface errors. Compared with traditional uniform smoothing, the proposed method achieves significant improvements across key surface quality metrics, including PV, RMS, and PSD. The experimental findings demonstrate that by adaptively modulating dwell time based on local gradient and curvature features, the proposed model enables more accurate error targeting and superior smoothing convergence.

## 5. Conclusions

In this study, a novel morphology gradient-aware spatiotemporal coupled smoothing model was proposed for the precision smoothing of optical components with MSF errors. The model integrates surface gradient and curvature characteristics into a dwell-time modulation framework and incorporates convolution-based material removal modeling to achieve localized and adaptive smoothing control. Simulation and experimental results consistently validate the effectiveness of the proposed method. The main conclusions are summarized as follows:

(1) A gradient- and curvature-driven modulation mechanism was established, enabling dynamic and region-specific dwell-time adjustment based on local surface morphology.
(2) The proposed model effectively reduces PV and RMS errors while achieving superior suppression of MSF features, as evidenced by significant improvements in PSD curve behavior.
(3) Compared to traditional uniform smoothing, the proposed approach demonstrates higher convergence rates, thereby enhancing final surface quality.

Furthermore, the proposed morphology-aware polishing model, though developed for optical surface smoothing, holds potential for extension to other flexible-tool-based finishing processes, such as magnetorheological finishing and elastic grinding, where surface-dependent pressure modulation plays a key role.

In future work, we will focus on extending the model to complex freeform optical surfaces, incorporating real-time measurement feedback, and developing closed-loop smoothing control strategies to further enhance adaptability and process automation.

## Figures and Tables

**Table 1:** Experimental setup and processing conditions.

Experimental Item	Specification
Robot system	Stäubli TX200
Component properties	Φ140 mm Fused silica
Smoothing tool	pitch pad
Polishing path	spiral path
Line spacing/point spacing	1/1 (mm)
Ambient temperature	25 °C
Rotation/revolution	120/100 (r/min)
Smooth pressure	15 (N)
Slurry	CeO_2_ (0.5 μm) (free concentration, flow maintained)

## Data Availability

The data presented in this study are available on request from the corresponding author due to institutional confidentiality agreements.
